# Neonatal Meningitis by Multidrug Resistant *Elizabethkingia meningosepticum* Identified by 16S Ribosomal RNA Gene Sequencing

**DOI:** 10.1155/2014/918907

**Published:** 2014-02-09

**Authors:** V. V. Shailaja, Ashok Kumar Reddy, M. Alimelu, L. N. R. Sadanand

**Affiliations:** ^1^Department of Microbiology, Niloufer Hospital for Women and Children, Hyderabad 500004, India; ^2^GHR Micro Diagnostics, Hyderabad 500082, India; ^3^Department of Pediatrics, Niloufer Hospital for Women and Children, Hyderabad 500004, India

## Abstract

Clinical and microbiological profile of 9 neonates with meningitis by *Elizabethkingia meningosepticum* identified by 16S ribosomal gene sequencing was studied. All the clinical isolates were resistant to cephalosporins, aminoglycosides, trimethoprim-sulfamethoxazole, **β**-lactam combinations, carbapenems and only one isolate was susceptible to ciprofloxacin. All the isolates were susceptible to vancomycin. Six of nine neonates died even after using vancomycin, based on susceptibility results. *E. meningosepticum* meningitis in neonates results in high mortality rate. Though the organism is susceptible to vancomycin in vitro, its efficacy in vivo is questionable and it is difficult to determine the most appropriate antibiotic for treating *E. meningosepticum* meningitis in neonates.

## 1. Introduction

*Elizabethkingia meningosepticum* (formerly known as *Chryseobacterium meningosepticum/Flavobacterium meningosepticum*) is a nonfermentative gram negative bacillus, ubiquitous in nature [1, 2]. *E. meningosepticum* causes meningitis, pneumonia, bacteremia, and sepsis in infants and pneumonia, endocarditis, postoperative bacteremia, and meningitis in adults [1–3]. Among the different infections, a high mortality and severe postinfection sequelae including hydrocephalus, deafness, and developmental delay have been reported in neonates with meningitis due to *E. meningosepticum* [2]. Infections caused by* E. meningosepticum* are difficult to treat because of its resistance to extended spectrum *β*-lactam agents and aminoglycosides [1–3]. In this study we report the clinical profile, antibiotic susceptibility, and treatment outcome of meningitis caused by multidrug resistant *Elizabethkingia meningosepticum* in neonates.

## 2. Materials and Methods

Nine neonates with *E. meningosepticum* meningitis who presented to the Neonatal Intensive Care Unit of Niloufer Hospital for Women and Children, Hyderabad, India, between January 2009 and December 2010, were included in the study. Cerebrospinal fluid collected from the neonates was subjected to direct microscopic examination, inoculated on to blood agar, chocolate agar, and MacConkey's agar, and incubated overnight at 37°C. Blood cultures were done by automated blood culture systems (Bact/Alert, Biomerieux, France). The isolates were identified by conventional biochemical reactions and Vitek 2 (Biomerieux, France). The identity of the isolates was further confirmed by 16S rRNA gene sequencing. Sequencing was performed (forward primer, 5′-TTGGAGAGTTTGATCCTGGCTC-3′; reverse primer, 5′-GGACTACCAGGGTATCTAA-3′) with fluorescence-labeled dideoxynucleotide terminators using an ABI 3130 Xl automated sequencer, following the manufacturer's instructions (PE Applied Biosystems). The sequences were analysed and identified using the Megablast search program of the GenBank database. The sequence of the isolate perfectly (100%) matched the sequences of *E. meningosepticum* deposited in GenBank. The gene sequences of the isolates were deposited in the GenBank. Antibiotic susceptibility of the isolates was done by Kirby Bauer's disk diffusion method and also by Vitek 2.

## 3. Results and Discussion

Gram's stain of CSF showed polymorphonuclear leukocytes and Gram negative bacilli in all the nine patients. Confluent growth of moist raised colonies was seen on blood and chocolate agar in all patients. Blood culture was collected in only one patient apart from CSF culture. Blood culture was positive and showed the growth of moist raised colonies on subculture on blood and chocolate agar. There was no growth on MacConkey's agar in all patients. All the isolates were non motile, and catalase and oxidase positive, indole positive, citrate and urease negative, ortho-nitrophenyl-beta-galactoside positive, gelatin was liquefied after 48 hours; oxidative fermentative test was positive after 72 hours. All the isolates were identified as *E. meningosepticum* by Vitek 2. Antibiotic susceptibility and GenBank accession numbers of the isolates were shown in Table 1. Clinical features and treatment outcome of the nine patients with *E. meningosepticum* meningitis are shown in Table 2.

The data on antibiotic susceptibility of *E. meningosepticum* is limited because it is rarely isolated from clinical specimen and there are no standard guidelines on antibiotic susceptibility testing and reporting and interpretation of the susceptibility data. According to the published literature *E. meningosepticum* is known to be resistant to *β*-lactams, extended spectrum cephalosporins, carbapenems, and gentamicin, susceptible to vancomycin, trimethoprim-sulfamethoxazole, rifampicin, and ciprofloxacin, and moderately susceptible to piperacillin [2]. In the present study we have noted that all the isolates are resistant to *β*-lactams, extended spectrum cephalosporins, *β*-lactam combinations, carbapenems, aztreonam, aminoglycosides, tetracyclines, and trimethoprim-sulfamethoxazole and only one isolate has been susceptible to ciprofloxacin. All the isolates are susceptible to vancomycin by disk diffusion method. Determination of antibiotic susceptibility by disk diffusion is not a recommended method, but there is no option in Vitek to check the susceptibility of nonfermenting Gram negative bacilli to vancomycin. There is no discrepancy between the susceptibility results of the isolates by Vitek and disk diffusion to the remaining antibiotics. Antibiotic susceptibility data of our study highlights that the majority of isolates (8/9) are multidrug resistant (resistant to all drugs except vancomycin).

The underlying host factors associated with *E. meningosepticum* meningitis in neonates are prematurity and low birth weight and we also observed the same risk factors [4, 5]. Based on the susceptibility of the isolates only to vancomycin, we have used vancomycin in 7/9 neonates and one neonate received combination of vancomycin and ciprofloxacin antibiotics. One neonate succumbed before the susceptibility results were available. Only two neonates recovered from infection after using vancomycin and the remaining 7 expired. According to the previous reports vancomycin is not an effective antimicrobial agent to treat the *E. meningosepticum* meningitis [2, 6, 7]. Based on our study findings and other reports the efficacy of vancomycin in treating *E. meningosepticum* meningitis in neonates is questionable. Previous authors treated neonates with *E. meningosepticum* meningitis successfully with piperacillin in combination with rifampicin [5]. All our study isolates were resistant to piperacillin and piperacillin-tazobactam. The limitation of our study is that susceptibility to rifampicin is not determined. The mortality rate (6/9) in our study is high compared to other studies [5].

Several outbreaks of *E. meningosepticum* meningitis in neonates have been reported [8, 9]. The outbreaks reported in the literature occurred within few weeks to months except in one study, where the authors reported an outbreak with one strain of *E. meningosepticum* for 2 years [8]. All the nine cases reported in the present study occurred over a period of one year. We have tried to identify the source of infection in the present study by collecting environmental specimens like water from incubators, tap water, suction fluids, the disinfectants and healthy babies were also screened for asymptomatic carriage by collecting rectal and umbilical swabs. All the environmental specimens and healthy babies were negative for *E. meningosepticum*. We also carried out the gene sequencing of the isolates from seven patients and phylogenetic tree was constructed to see the genetic relatedness of the isolates (gene sequences of the isolates deposited in the GenBank) and found that none of the isolates are genetically related to each other. In the present study we could not identify the source of infection.

## 4. Conclusion 

*E. meningosepticum* meningitis in neonates results in high mortality rate. Though the organism shows susceptibility in vitro to vancomycin, its efficacy in vivo is questionable and it is difficult to determine the most appropriate antibiotic for treating *E. meningosepticum* meningitis in neonates.

## Figures and Tables

**Table 1:** Antibiotic susceptibility and GenBank accession numbers of isolates.

S. number	GenBank accession number	Antibiotic susceptibility								
		AK/G	CIP/OF	CZ/CTX/CFP	IM/MP	PC	PTB	AC	V	SXT
1	HM042305	R	R	R	R	R	R	R	S	R
2	HM042306	R	S	R	R	R	R	R	S	R
3	HM130055	R	R	R	R	R	R	R	S	R
4	HM130056	R	R	R	R	R	R	R	S	R
5	HM130057	R	R	R	R	R	R	R	S	R
6	HM130058	R	R	R	R	R	R	R	S	R
7	HM130059	R	R	R	R	R	R	R	S	R
8	NS	R	R	R	R	R	R	R	S	R
9	NS	R	R	R	R	R	R	R	S	R

PC: piperacillin; PTB: piperacillin-tazobactam; AK: amikacin; G: gentamicin; CZ: ceftazidime; CTX: cefotaxime; CFP: cefepime; MP: meropenem; IM: imipenem; CP: ciprofloxacin; OF: ofloxacin; SXT: trimethoprim-sulfamethoxazole; V: vancomycin; R: resistant; S: sensitive; NS: not submitted in the GenBank.

**Table 2:** Clinical features and treatment outcome of nine neonates with meningitis.

S. number	Age (days)/sex	Underlying condition	Clinical features at presentation	Clinical diagnosis	Initial antibiotic used before collection of specimen	Modified antibiotic therapy after microbiological diagnosis	Outcome
1	2/F	Preterm baby	Fever, seizures	Meningitis	Cefotaxime and amikacin	Vancomycin	Recovered

2	17/F	Preterm baby	Seizures, neck rigidity	Meningitis	Cefotaxime and amikacin	Vancomycin and ciprofloxacin	Recovered

3	15/F	Preterm baby	Fever, icterus, seizures	Septicemia and meningitis	Cefotaxime and Amikacin	Vancomycin	Left against medical advise

4	7/F	Low birth weight	Seizures, icterus, vomiting	Septicemia	Cefotaxime and amikacin	Vancomycin	Expired

5	21/M	Low birth weight, preterm baby	Seizures, fever, neck rigidity	Septicemia and meningitis	Cefotaxime and amikacin	Vancomycin	Expired

6	16/F	Low birth weight, preterm baby	Seizures, fever, neck rigidity	Meningitis	Cefotaxime and amikacin	Vancomycin	Expired

7	30/M	Low birth weight, preterm baby	Seizure, vomiting, neck rigidity	Meningitis	Cefotaxime and amikacin	Vancomycin	Expired

8	22/M	Preterm baby	Seizure, neck rigidity	Meningitis	Cefotaxime and amikacin	Vancomycin	Expired

9	10/F	Low birth weight, preterm baby	Not accepting feeds, seizures, neck rigidity	Meningitis	Cefotaxime and amikacin	Expired before the susceptibility results were available	Expired
